# Visualizing Cortical Development and Evolution: A Toolkit Update

**DOI:** 10.3389/fnins.2022.876406

**Published:** 2022-04-12

**Authors:** Takuma Kumamoto, Chiaki Ohtaka-Maruyama

**Affiliations:** Developmental Neuroscience Project, Department of Brain and Neurosciences, Tokyo Metropolitan Institute of Medical Science, Tokyo, Japan

**Keywords:** neuronal labeling, cortical development, cortical evolution, visualizing tool, somatic transgenesis

## Abstract

Visualizing the process of neural circuit formation during neurogenesis, using genetically modified animals or somatic transgenesis of exogenous plasmids, has become a key to decipher cortical development and evolution. In contrast to the establishment of transgenic animals, the designing and preparation of genes of interest into plasmids are simple and easy, dispensing with time-consuming germline modifications. These advantages have led to neuron labeling based on somatic transgenesis. In particular, mammalian expression plasmid, CRISPR-Cas9, and DNA transposon systems, have become widely used for neuronal visualization and functional analysis related to lineage labeling during cortical development. In this review, we discuss the advantages and limitations of these recently developed techniques.

## Introduction

Neuroscientists for the longest time have been fascinated by the idea of neocortex as the “seat of intelligence,” tasked with integrating sensory inputs and supporting higher order cognitive processes that are paramount for survival. In the neocortex of the members of the mammalian clade, billions of neurons of various subtypes are organized radially into a stereotypical six-layered cytoarchitecture and tangentially into functional areas that subserve different cognitive functions (Cadwell et al., 2019). The way in which these structures relate to the neocortical functions, the developmental process underlying its formation, and the evolution of the neocortices of not just the mammals but the closely related sauropsids, are critical questions that have yet to be fully answered.

Tools that label or allow for the genetic manipulation of neurons proved to be invaluable in this respect, providing information about a neuron’s birthdate and lineage at the time of labeling. The simplest form of labeling would be to utilize fluorescent proteins or small peptide tags to visualize a specific type of neurons. In its current iteration, this would be done using transgenic animals; reporter lines expressing “labeling” proteins such as LacZ and other fluorescent proteins (e.g., green fluorescent protein (GFP), red fluorescent protein (RFP), yellow fluorescent protein (YFP); hereafter referred to as XFPs), are crossed with driver lines expressing recombinases under the promoters of the specific gene of interest to selectively label target cell types (Soriano, 1999; Srinivas et al., 2001; Madisen et al., 2010). The explosion in the number of commercially available transgenic mice lines (Taniguchi et al., 2011; Abe and Fujimori, 2013; Madisen et al., 2015; Daigle et al., 2018), the large amount of information on them on the databases of Mouse Genome Informatics (MGI)^1^ and the Allen Institute for Brain Science^2^ is testament to its popularity and success as a tool amongst neuroscientists using mouse as a model organism. However, as the field progresses, the usefulness of the house mouse (*Mus musculus*) as a prime model organism begins to fade in favor of other newly developed ones. This is in part due to its limitation as a lissencephalic mammal, but more so because evo-devo neuroscientists need to work on multiple organisms to elucidate the evolutionary mechanisms underlying neurogenesis. To establish transgenic lines in all the new model animals would be costly and time-consuming, or even out of the question in the case of human translational research on cognitive disorders. Hence there is a need for genetic manipulation tools that allow researchers to conduct their experiments without the use of transgenic lines, but also provide appropriate level of temporal and spatial control over the neurons that are targeted.

**FIGURE 1:**
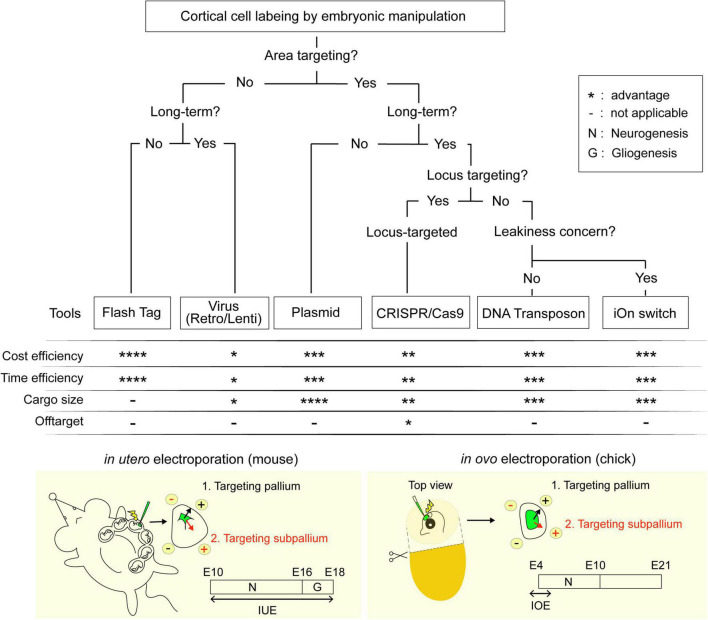
Flowchart for the selection of labeling techniques. Techniques are ranked from * indicating least advantageous to **** indicating the most advantageous within the respective categories.

Current approaches for neuronal visualization include the introduction of chemicals or vectors for endogenous genetic material, such as viruses or plasmids, into the cerebral ventricle (Figure 1). This specifically targets neural progenitors residing in the ventricular zone (VZ), and consequently all neurons born from them. As cortical neurons are generated sequentially in an inside-to-outside pattern, temporal control over the neuronal subtype being labeled can be achieved by varying the timing of injection. When combined with in utero electoroporation (IUE) [or in some cases in Ovo electroporation (IOE)], where applicable, researchers are able to obtain further spatial control over the cortical areas being targeted (Figure 1, bottom). Since it was first described in 2001 (Fukuchi-Shimogori and Grove, 2001; Saito and Nakatsuji, 2001; Tabata and Nakajima, 2001), IUE/IOE has been used to successfully introduce exogenous plasmids into the cortices of not only mice but also chicks (Nomura et al., 2018), quails (Nomura et al., 2008), turtles (Nomura et al., 2013), geckos (Nomura et al., 2013), snakes (Cárdenas et al., 2018; Cárdenas and Borrell, 2021), and ferrets (Borrell, 2010; Chinnappa et al., 2022), underscoring the importance of IUE/IOE as a tool to understanding cortical development and evolutionary mechanisms. While the process of electroporation remains largely unmodified, advancements in genetics have led to the development of plasmids that can be tailored to the specific requirements of each experiment. This review aims to highlight these creative ideas, not just to inform but in hopes that they can be incorporated by colleagues working on model organisms that are still in need for experimental tools.

## Main

### Injection

#### Labeling Using Chemicals

Injection of thymidine analogs such as bromodeoxyuridine (BrdU) and 5-ethynyl-2′-deoxyuridine (EdU), allows the labeling of actively dividing progenitors at the time of injection. As the thymidine analogs are diluted with each successive cell division, only neurons born immediately following the injection will be strongly labeled. The recently developed Flash Tag (FT) method further limits labeling to only the neural progenitor population, by taking advantage of the process of interkinetic nuclear migration (IKNM) in which VZ progenitors move to the ventricular surface during the M-phase of the cell cycle. In contrast to previous methods of injections, FT introduces carboxyfluorescein esters (CFSEs) dyes, which only fluoresces when taken up by cells, into the ventricle of the mouse embryo. With a short labeling time of 1–2 h, FT is a much more powerful tool for fate mapping, birthdate analysis, and migration analysis as compared to its predecessors (Telley et al., 2016; Govindan et al., 2018; Yoshinaga et al., 2021). However, as FT labels cells along the entire ventricular wall, it may be less suited for experiments which require more spatial control over the cells being targeted.

#### Labeling Using Viral Vectors

Intraventricular viral labeling using retrovirus (including lentiviruses) has been established over 30 years ago (Price, 1987; Cepko et al., 2000), and has contributed to numerous landmark papers in the field of developmental neuroscience (Golden et al., 1995; Szele and Cepko, 1996; Cepko et al., 2000). Its main advantage over chemical labeling is that viruses are able to integrate the respective GOIs into the genome of the host cell randomly then express it permanently without dilution by cell division; while on its own it provides limited spatial control, the shortcomings can be easily overcome by using it in conjunction with site-specific driver mice (Ciceri et al., 2013). This limits its use to sites in which transgenic lines are established, but where these lines are available, the low-titer injection of replication-deficient retrovirus reporters into transgenic mice expressing a tissue-specific recombinase driver remains a popular method for clonal analysis (Llorca et al., 2019). Multiplex clonal labeling provides a different alternative, generating unique “tags” which can be tracked in parallel to analyze a cell’s lineage. Here, the recently established lentiviral gene ontology (LeGO) method utilizes three different fluorescent proteins (Red, Green, and Blue) expressed at two different intensities to generate up 26 unique color-codes which can be sorted by multiplex flow cytometry. These vectors were shown to integrate and be expressed stably after several rounds of cell division, and thus are suitable for both *in vitro* and *in vivo* analysis (Weber et al., 2011).

### Electroporation

#### Labeling Using DNA Plasmids

As it provides a decent amount of temporal and spatial control over the neurons being targeted, IUE is by far the most popular technique for genetic manipulation. Transfection is achieved in a two-step process: (1) Microinjection of plasmids containing the gene of interest (GOI) into the ventricular lumen, (2) followed by the delivery of electrical pulses facilitated by an extra-uterine pair of electrodes. The electrical pulses destabilize the lipophilic membrane of the progenitors present at the ventricular surface, and negatively charged DNA is guided into cells closer to the anode end of the electrodes. Thus, different cortical areas can be targeted by changing the angle at which the electrodes are placed (Szczurkowska et al., 2016). Commercially available mammalian expression plasmids are mostly designed for the transient episomal expression of GOIs in mammalian cells without genome insertion. Common approaches utilize a site-specific recombinase (SSR), expressed either endogenously under the control of cell or tissue specific promoters or co-electroporated with reporter plasmids such as the Cre/*loxP* and FLEx systems (Schnütgen et al., 2003). A key point to note is that the high sensitivity of the SSR may lead to unwanted recombination (a concentration of 0.8 nM Cre recombinase is sufficient to recombine reporter) (Ringrose et al., 1998). As a result, episomal expression from electroporated plasmids remaining in the postmitotic cells even after several rounds of differentiation may cause unwanted recombination and confound the results from lineage analysis (Schick et al., 2019).

Currently two different approaches have been employed to achieve a higher cellular single cell resolution. The Tetbow system introduces a mixture of XFP plasmids into the cells resulting in stochastic multi-color labeling of electroporated neurons based on the combinatorial expression of XFPs (Sakaguchi et al., 2018). The switch to a tetracycline trans-activator system has further boosted the expression of XFPs, markedly improving the signal to noise ratio as compared to its previous iteration, brainbow (Livet et al., 2007). In addition, the authors have developed a chemical-tagged version of tetbow that can be used to label neuronal axons with multicolor, making it an ideal tool for elucidating the mechanisms of neuronal circuit formation (Sakaguchi et al., 2018). The Supernova technique on the other hand utilizes the low leakiness of the tetracycline response element (TRE) for sparse labeling of neurons (Luo et al., 2016). This initial weak expression is then enhanced by tTA/TRE positive feedback, resulting in the bright labeling of neuronal cells for the visualization of morphological features.

The myriad of plasmids and tools available allow for flexible genetic manipulation that can be tailored to suit the needs of the experiment. Owing to the rapid dilution of non-integrated expression vectors during cell division in the cortical progenitors, this method is preferably used for time-stamped neuronal birthdate labeling and the analysis of neuronal migration patterns.

#### Labeling Using the CRISPR-Cas9 System

Integration of the gene of interest into the genome of the target cell resolves the technical problem of transient expression. Locus specific insertion can be achieved electroporation of the CRISPR-Cas9 system. A double stranded break is created at the location specified in the guide RNA (gRNA) and the supplied sequence of interest can be integrated either by non-homologous end joining (NHEJ) or homology-directed repair in post-mitotic cells and actively dividing progenitors, respectively. Since its introduction as a tool for genetic manipulation, it has undergone several rounds of modification to overcome some of the early problems. For example, Tsunekawa et al. (2016) constructed the *pLeakless-III* vector which quenches donor leakage prior to insertion. To increase the insertion efficiency, the single-cell labeling of endogenous proteins by CRISPR-Cas9-mediated HDR” (SLENDR) (Mikuni et al., 2016) technique switched to utilizing single-stranded oligodeoxynucleotides (ssODNs)-based donor, as well as drastically reducing the size of the insert length by using small epitope tags. The race to optimizing the technique is still on-going with Targeted Knock-In with Two (TKIT) guides (Fang et al., 2021) and Brain Easi-CRISPR (Breasi-CRISPR) (Meyerink et al., 2022) being published recently.

#### Labeling Using Transposons

##### Classic Transposon Approach

Despite the improvements in efficiency, where benefits of targeted genomic insertion brought about by the CRISPR-Cas9 approach are not necessary, researchers might prefer to opt for the transposon approach. The classic transposon approach utilizes the naturally occurring class II transposable elements as a vehicle to integrate DNA into the target cell via a “cut and paste” mechanism. Plasmids containing the sequence of interest flanked by terminal inverted repeats (TIRs) can be electroporated, and when mobilized by the appropriate transposase supplemented *in trans*, results in the stable and efficient integration into the target cell genome. The rise in the popularity of transposon based approaches in favor of transgenic animal lines in numerous *in vivo* applications, including fate mapping (Chen and LoTurco, 2012), multiplex clonal labeling (García-Marqués and López-Mascaraque, 2013; García-Moreno et al., 2014; Loulier et al., 2014), assessment of gene function (Serralbo et al., 2013), or direct screening of genes involved in developmental processes (Lu et al., 2018), is not only due to its simplicity and high integration rates, but also the tolerance for large cargoesup to 100 kb (Li et al., 2011). This allows for insertion of a lineage specific promoter in addition to a reporter protein, and is extremely useful for the visualization of cells which cannot be efficiently accessed by IUE at their birthdate owing to technical difficulties. For example, visualization of astroglial cells which was done conventionally by postnatal electroporation, can now be was carried out much more effectively by introducing GFP expressed under the astroglial-specific promoter using the transposon approach (Hamabe-Horiike et al., 2021). Star Track takes this approach one step further by introducing a mixture of six different FPs under the control of GFAP (astrocyte) (García-Marqués and López-Mascaraque, 2013), Ubc (ubiquitous) (Figueres-Oñate et al., 2016), and NG2 (oligodendrocyte) (Sánchez-González et al., 2020) promoters for multi-color stochastic labeling of astroglial cells for lineage analysis. Other strategies such as multi addressable genome-integrative color (MAGIC) markers (Loulier et al., 2014) and clonal labeling of neural progenies (CLoNe) (García-Moreno et al., 2014) opt for the use of SSR-dependent reporter tools to generate stochasticity. Currently, these aforementioned tools use, or have variations that are compatible with one of the two more popular transposes, piggyBac and Tol2, and should be applicable to multiple animal model species. More interestingly the CRISPR-Cas9 system combined with piggyBac transposon was utilized to help visualize the changing state of progenitor cells over the course of development. Temporal Encoding and Manipulation in a Predefined Order (TEMPO) developed by the Lee Lab functions by an irreversible sequential activation of FPs by a cascade of gRNAs expressed in parallel (Espinosa-Medina et al., 2021). When introduced into cortical progenitors, it labels post-mitotic neurons born sequentially based on the FPs expressed at the particular time in neurogenesis. To date, it is the only tool in this regard, and shows great promise in functioning as a potential cell cycle counter in progenitors over the course of neurogenesis.

##### Integration-Coupled Gene Expression on (iOn) Switch

**FIGURE 2:**
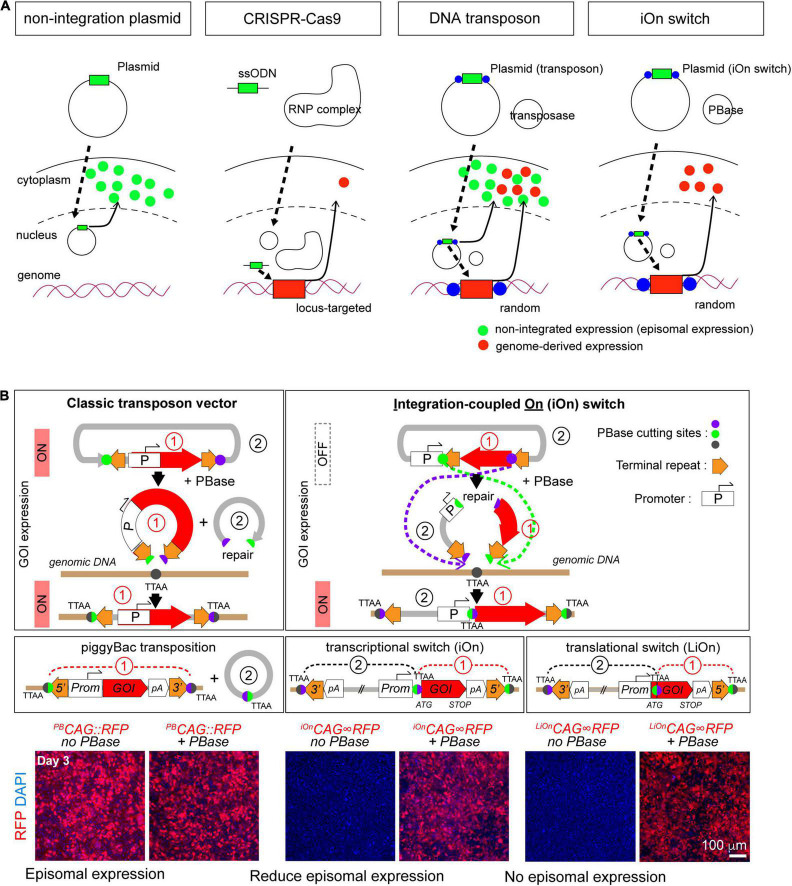
Principle of the electroporation-based gene transfer tools. **(A)** Comparison of the gene transfer mechanisms. **(B)** Principle of gene transfer with classic transposon (left) and iOn switch (right). GOI: gene of interest. Images used from Kumamoto et al. (2020), Neuron according to copyright.

The classic transposon approach has always been faced with the challenge of differentiating between expression of genes of interest from genomic insertions and residual episomal copies of the transgene (Figures 2A,B). Transient expressions can not only result in leakage from lineage specific promoters, they also confound lineage analyses as they expression prevent markers from acting as a reliable readout of the integrated transgenes. To address this problem we developed a completely new type of genome integration-coupled genetic switch, which we named “iOn switch” (Kumamoto et al., 2020). The iOn switch completely eliminates episomal expression before integration into the genome by (1) separating the promoter and target gene and placing them in opposite directions, and (2) placing the two transposon response elements in the same orientation between the promoter and the target gene (Figure 2B). iOn switch’s high integration rates mediated by the piggyBac transposase, and the simplicity of its design allows it to be used in both conventional transfection and IUE, making it a viable strategy for both *in vitro* culture experiments and IUE in various animal models. For example, our data showed that iOn switch has a similar transfection and integration efficiency in vitro when compared to the classic piggyBac-only approach. In addition, our *in vivo* data also confirmed similar integration kinetics between the two. Our application of iOn switch to the determination of clonal output of progenitor subtypes in the developing chick retina, as well as functional mosaic analysis in the study of homeostatic control of neurogenesis in the embryonic chick neural tube further demonstrates the versatility of the technique as an approach to the current developmental questions (Kumamoto et al., 2020). As an effort to extend the tool to other model organisms, we are currently using the iOn switch to conduct lineage and clonal analysis in avian and reptilian brains, as well as comparative functional mosaic analysis to study the cell autonomous and non-autonomous functions to provide new insights to our current understanding of evolution. Given its huge potential in the facilitation of genetic manipulations, we believe that its use will be indispensable for future studies in evolution and neurodevelopment.

## Perspectives and Concluding Remarks

It is undeniable that our understanding of neocortical neurogenesis has improved by leaps and bounds in the recent decade. As the neuroscientists begin to explore the use of new *in vitro* and *in vivo* models the study of development of gyrencephalic brains, human diseases and the evolution of the neocortex, the development of new tools for visualization and genetic manipulation become even more important than before. The flowchart we have created in Figure 1 covers all the different tools reviewed in this paper in hopes of aiding colleagues in choosing the best technique for their specific research question.

Amongst the various techniques presented, we believe that IUE would be the best way forward as it provides both spatial and temporal control over the neurons being target. IUE, however, does have its technical limitations, most of them concerning the visibility and accessibility to the ventricular lumen. For example, IUE of mice becomes difficult perinatally; despite having a neurogenic period up till E10, IOE in the chick can only be performed between E3 and E5 as the maturation of the blood vessels and the eye prevents access to the neocortex without damaging surrounding tissue (Figure 1, bottom). This can, in theory, be circumvented using a transposon-based approach similar to that used by Hamabe-Horiike et al. (2021). Alternatively, genetic manipulation can be done using the improved-Genome editing *via* Oviductal Nucleic Acids Delivery (i-GONAD) system, which achieves the CRISPR-Cas9-based transfer of ssODNs to E0.7 embryos capable of generating of knock-out by indels and knock-in of up to 1 kb in length (Ohtsuka et al., 2018). Other mammals such as the marmoset have thick placentas that obscures the view of the embryo. In a similar vein, researchers targeting the mice subplate neurons born around E10 face a similar difficulty localizing the embryo (Ohtaka-Maruyama et al., 2018). The ultrasound guided electroporation approach used in Neural Plate Targeting within Utero Nano-Injection (NEPTUNE) for lentiviral injections at E7.5, could be modified for IUE to aid in localizing the ventricles (Mangold et al., 2021). Given the popularity of IUE as a technique, it is no wonder that improvements to the technical aspects of IUE, as well as the tools driving exogenous expression of the GOI are being developed at such breakneck speeds. We strongly believe that it is a matter of time before IUE will be well adapted for gene manipulation in the relatively newer models of neurogenesis.

## Author Contributions

TK drafted the manuscript and created the Figures. TK and CO-M discussed and revised the manuscript. Both authors contributed to the study and approved the final manuscript.

## Conflict of Interest

The authors declare that the research was conducted in the absence of any commercial or financial relationships that could be construed as a potential conflict of interest.

## Publisher’s Note

All claims expressed in this article are solely those of the authors and do not necessarily represent those of their affiliated organizations, or those of the publisher, the editors and the reviewers. Any product that may be evaluated in this article, or claim that may be made by its manufacturer, is not guaranteed or endorsed by the publisher.

## Footnotes

1
http://www.informatics.jax.org/

2
https://connectivity.brain-map.org/transgenic
